# EpiToolKit—a web-based workbench for vaccine design

**DOI:** 10.1093/bioinformatics/btv116

**Published:** 2015-02-20

**Authors:** Benjamin Schubert, Hans-Philipp Brachvogel, Christopher Jürges, Oliver Kohlbacher

**Affiliations:** ^1^Center for Bioinformatics, University of Tübingen, 72076 Tübingen, Germany, ^2^Applied Bioinformatics, Department of Computer Science, 72076 Tübingen, Germany, ^3^Quantitative Biology Center, 72076 Tübingen, Germany and ^4^Faculty of Medicine, University of Tübingen, 72076 Tübingen, Germany

## Abstract

**Summary:** EpiToolKit is a virtual workbench for immunological questions with a focus on vaccine design. It offers an array of immunoinformatics tools covering MHC genotyping, epitope and neo-epitope prediction, epitope selection for vaccine design, and epitope assembly. In its recently re-implemented version 2.0, EpiToolKit provides a range of new functionality and for the first time allows combining tools into complex workflows. For inexperienced users it offers simplified interfaces to guide the users through the analysis of complex immunological data sets.

**Availability and implementation:** http://www.epitoolkit.de

**Contact:** schubert@informatik.uni-tuebingen.de

**Supplementary information:** Supplementary data are available at *Bioinformatics* online.

## 1 Introduction

Epitope-based vaccine design offers novel and rational ways to develop vaccines based on genomic information. The design process undergoes several steps. The first step aims at identifying antigenic peptides (called epitopes) that induce a T-cell mediated immune reaction after presentation on the cell surface by proteins of the major histocompatibility complex (MHC). In the second step a subset, usually of size 10–20 epitopes, is selected forming the basis of the vaccine. Due to the high polymorphism within the MHC cluster, each individual possesses a unique set of MHC alleles and therefore presents a different set of epitopes. Hence, it is not only necessary to identify the individuals MHC genotype but also to tailor the epitope selection to match the MHC allele restrictions of a population (population-optimized vaccine) or that of an individual (personalized vaccines). The third step of the design pipeline is concerned with the delivery of the selected epitopes. A common strategy concatenates the epitopes into a so-called string-of-beads polypeptide. The epitope order within a string-of-beads plays a crucial role especially in degradation. Therefore it is necessary to optimize the ordering such that the recovery probability of the epitopes is maximal.

Since the underlying data and the interdependencies of the design pipeline are complex and require bioinformatics tools to obtain optimal results, we developed a web-based platform EpiToolKit (ETK) to make such approaches accessible to a broader audience. ETK extends its predecessor by supporting MHC genotyping, and epitope assembly besides epitope discovery and epitope selection. Thus, it covers each of the described design steps and can be used for personalized or population-optimized vaccine development as well as for other immunological applications (e.g. large-scale epitope prediction). Additionally, functionalities such as the supported prediction methods and input formats have been extended. Also ETK is now based on a customized version of the open-source platform Galaxy (Goecks *et al.*, 2010), which allows a flexible combination of tools into workflows, a reliable recording and sharing of results, and the integration with high-performance computing resources.

## 2 Material and methods

ETK was designed to ease the use for inexperienced users but still retain high flexibility in combining the different tools. Under the tab *Single Tools* the interfaces are simplified into several configuration steps equipped with help texts. Under the *Workflow* tab these steps are available as independent nodes, allowing the development of complex workflows. All ETK tools generate two outputs: an interactive presentation of the results as html and an internal representation that can be used as input to other tools.

ETK integrates OptiType, a newly developed NGS-based MHC genotyping approach that is superior in accuracy to existing methods (Szolek *et al.*, 2014). OptiType uses integer linear programming to simultaneously select all major MHC class I alleles comprising the genotype and supports Exome-Seq, RNA-Seq and whole-genome sequencing data. ETK also provides access to a collection of popular epitope prediction tools. The available methods include SYFPEITHI, BIMAS, SVMHC, the NetMHC family (reviewed in Toussaint and Kohlbacher, 2009), UniTope (Toussaint *et al.*, 2011a), and TEPITOPEpan (Zhang *et al.*, 2012). With *Polymorphic Epitope Prediction* ETK extends epitope prediction to the incorporation of sequencing variations and is therefore vital for personalized design approaches. This method is based on SNEP (Schuler *et al.*, 2005) and was extended to handle indels and frame shifts besides single nucleotide polymorphisms. It either searches for known variations of a given protein within dbSNP (Sherry *et al.*, 2001) or uses a list of variations in vcf format. In both cases the variations are annotated using ANNOVAR (Wang *et al.*, 2010) to construct all polymorphic epitopes. This pipeline can be used to identify minor histocompatibility antigens (Feldhahn *et al.*, 2012) or neoepitopes, which are of high interest in cancer vaccine design (Kyzirakos *et al.*, 2013).

For epitope selection ETK re-implements the mathematical framework OptiTope (reviewed in Toussaint and Kohlbacher, 2009). It determines a set of epitopes that maximizes the overall immunogenicity under constraints and thus the probability of inducing a long lasting immunity. Overall immunogenicity of an epitope set is defined as the sum over the immunogenicity of each epitope MHC allele pair, weighted by the probability of an MHC allele to appear in the target population or person.

The problem of epitope ordering for string-of-beads design has been previously formulated as a traveling salesman problem (Toussaint *et al.*, 2011b) and is now available in ETK. Since this approach is dependent on proteasomal cleavage site predictions, ETK offers two cleavage prediction approaches, PCM and NetChop (reviewed in Toussaint and Kohlbacher, 2009).

## 3 Applications

**Fig. 1.**
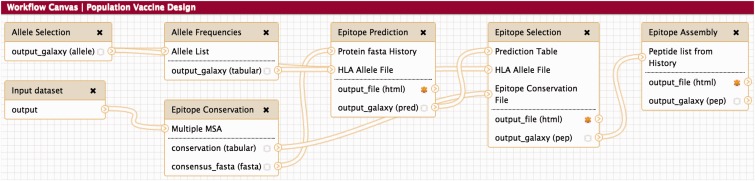
Example Workflow for population-based vaccine design. *Allele Selection* allows to specify the target population represented by their MHC alleles. *Allele Frequencies* then assigns frequencies to the chosen MHC alleles based on preassembled data or manually assigned frequencies. *Epitope Conservation* takes a file containing multiple MSA of antigens and constructs consensus sequences for each of them and calculates conservation scores for each k-mer peptide generated from the consensus sequences*. Epitope Prediction* performs the epitope prediction for the specified MHC alleles and the consensus sequences. *Epitope Selection* consumes the prediction results and selects a pre-defined number of epitopes under constraints for the specified target population and antigens. *Epitope Assembly* arranges the selected epitopes such that their recovery probability after proteasomal cleavage is maximal

To demonstrate ETKs capabilities, a workflow for designing population-optimized vaccines for seasonal influenza was developed (Fig. 1). Based on the yearly WHO recommendations a dataset consisting of H1N1 and H3N2 strains was extracted from the Influenza research database (Squires *et al.*, 2012). Using NetMHC and default configurations for the Epitope Selection step, 10 epitopes were selected. The epitopes covered 5 out of 10 antigens and 26 out of 47 MHC alleles with a population coverage of 99.66%. On average each epitope was predicted to bind to 14±3.3 MHC alleles. According to the Immune Epitope Database (Vita *et al.*, 2009) 10 out of 10 epitopes are known MHC binders or substrings of such and 5 out of 10 are T-cell reactive epitopes or substrings of such. For detailed results see Supplementary Tables S1–S3.

## 4 Conclusion

With ETK we provide a flexible and yet easy to use platform for rational vaccine design. Beyond the presented application ETK can be used to tackle a manifold of other immunological questions and thus should not only be valuable for applied medical but also for basic immunological research.

## Funding

This study was partially funded by DFG (SFB 685/B1, KO 2313/6-1) and BMBF (01GU1106).

*Conflict of Interest:* none declared.

## Supplementary Material

Supplementary Data
