# Supplementary material for: EpiToolKit—a web-based workbench for vaccine design
Source: Bioinformatics. 2015 Feb 20;31(13):2211–3. doi: 10.1093/bioinformatics/btv116 (PMC4481845; doi:10.1093/bioinformatics/btv116)
Supplement: Supplementary Data [file supp_31_13_2211__index.html]

EpiToolKit—a web-based workbench for vaccine design — EpiToolKit—a web-based workbench for vaccine design — Supplementary Data 

# EpiToolKit—a web-based workbench for vaccine design

## Supplementary Data

files

**Files in this Data Supplement:**

- Supplementary Data - zip file
