# Supplementary material for: EpiToolKit—a web-based workbench for vaccine design
Source: Bioinformatics. 2015 Feb 20;31(13):2211–3. doi: 10.1093/bioinformatics/btv116 (PMC4481845; doi:10.1093/bioinformatics/btv116)
Supplement: Supplementary Data [file supp_btv116_suppl_data.zip › supplementary.docx]

EpiToolKit – A Web-based Workbench for Vaccine Design – Supplementary Material

Benjamin Schubert^1,2,*^, Hans-Philipp Brachvogel^1^, Christopher Jürges^1^ and Oliver Kohlbacher^1,2,3,4^

^1^Center for Bioinformatics, University of Tübingen, 72076 Tübingen, Germany

^2^Applied Bioinformatics, Dept. of Computer Science, 72076 Tübingen, Germany

^3^Quantitative Biology Center, 72076 Tübingen, Germany

^4^Faculty of Medicine, University of Tübingen, 72076 Tübingen, Germany


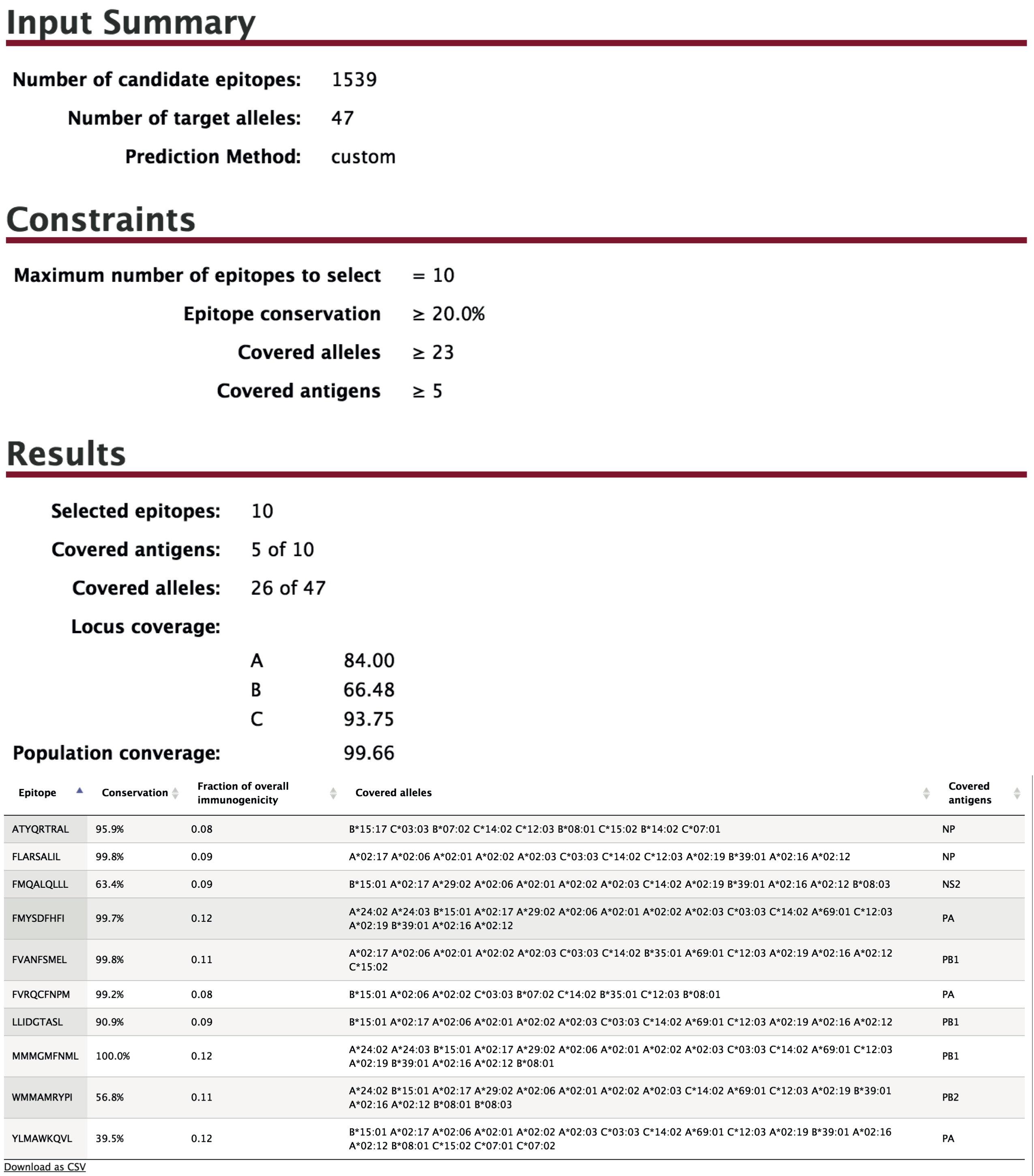


Supplementary Table 1. Epitope Selection for an influenza dataset consisting of H1N1 and H3N5 strain. The epitope set was optimized for the European population. NetMHC was used for epitope discovery and default constraints of 50% MHC allele and antigen coverage and 20% epitope conservation was used.


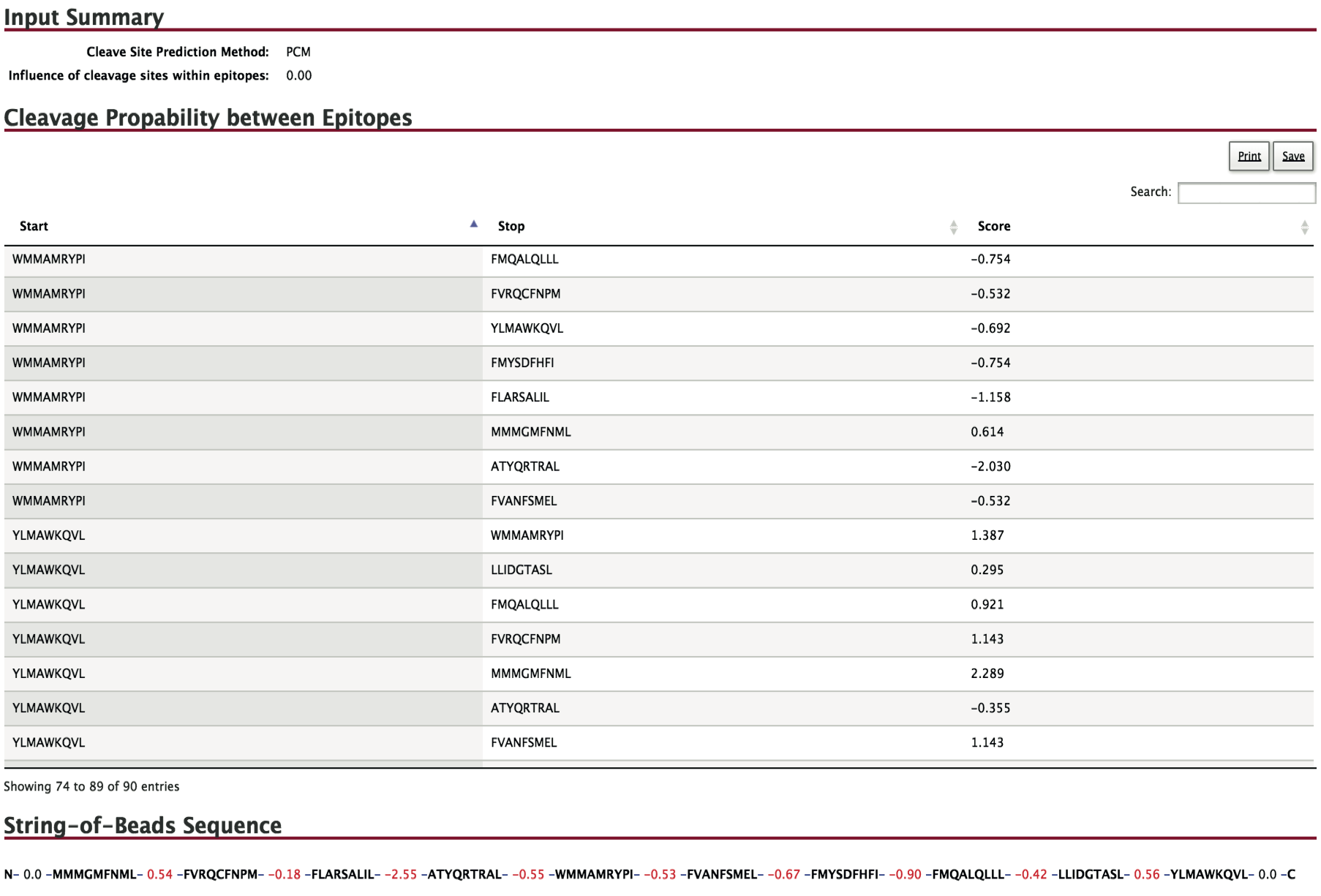


Supplementary Table 2. Epitope Assembly for the selected epitope set. PCM (default setting) was used as proteasomal cleavage prediction method. Harmful cleavage sites arising from the epitope ordering within epitopes were not punished.

Supplementary Table 3. Experimental evidence for the *in silico* predicted and selected epitopes and their corresponding IEDB IDs.

| Epitope | MHC-Ligand | T-Cell reactive | IEDB ID |
| --- | --- | --- | --- |
| FMYSDFHFI | Yes | Yes | 17119 |
| MMMGMFNML | Yes | Yes | 42143 |
| YLMAWKQVL | Yes |  | 124888 |
| FVANFSMEL | Yes | Yes | 97314 |
| WMMAMRYPI | Yes |  | 124859 |
| FLARSALIL | Yes | No | 16522 |
| FMQALQLLL | Yes |  | 178842 |
| LLIDGTASL | Subsequence | Subsequence | [129079](http://www.immuneepitope.org/epId/129079), 129607, [212044](http://www.immuneepitope.org/epId/212044), [218205](http://www.immuneepitope.org/epId/218205) |
| ATYQRTRAL | Yes | Subsequence | 5230, [7655](http://www.immuneepitope.org/epId/7655), [41793](http://www.immuneepitope.org/epId/41793), [79763](http://www.immuneepitope.org/epId/79763), [146073](http://www.immuneepitope.org/epId/146073), [164384](http://www.immuneepitope.org/epId/164384), [181194](http://www.immuneepitope.org/epId/181194) |
| FVRQCFNPM | Yes |  | 18274 |

# Source Code:

The source code of ETK-specific tools (Epitope Prediction, Polymorphic Epitope Prediction, Epitope Selection, and Epitope Assembly) can be found at GitHub:

https://github.com/b-schubert/Fred2/tree/feature/etk/

The code is provided *as is* and includes a ETK-specific pre-release of a larger framework. Several of the prediction methods are already included, others like the NetMHC-family have to be installed separately. The ETK command line tools can be found under Fred2/Apps/. For deployment into other system, the source code has to be slightly modified.

The source code for OptiType can be found here:

https://github.com/FRED-2/OptiType
